# p53 Mutant p53^N236S^ Induces Neural Tube Defects in Female Embryos

**DOI:** 10.7150/ijbs.31451

**Published:** 2019-07-25

**Authors:** Jinzhi Zhao, Yingbing Tian, Huihui Zhang, Lianhua Qu, Yu Chen, Qing Liu, Ying Luo, Xiaoming Wu

**Affiliations:** Laboratory of Molecular Genetics of Aging & Tumor, Medical School, Kunming University of Science and Technology, Chenggong Campus, 727 South Jingming Road, Kunming, Yunnan 650500, China

**Keywords:** p53^N236S^, neural tube defects, H3K27me3

## Abstract

The p53 is one of the most important tumor suppressors through surveillance of DNA damages and abnormal proliferation signals, and activation the cell cycle arrest and apoptosis in response to stress. However, the mutation of p53 is known to be oncogenic by both loss of function in inhibiting cell cycle progress and gain of function in promoting abnormal proliferation. In the present study, we have established a knock in mouse model containing an Asn-to-Ser substitution at p53 amino acid 236 by homologous recombination (p53N236S). Other than tumorigenesis phenotype, we found that *p53^S/S^*mice displayed female-specific phenotype of open neural tube in brain (exencephaly) and spinal cord (spina bifida). The occurrence rate for embryonic exencephaly is 68.5% in female *p53^S/S^* mice, which is much more than that of in *p53^-/-^* mice (37.1%) in the same genetic background. Further study found that p53^N236S^ mutation increased neuronal proliferation and decreased neuronal differentiation and apoptosis. To rescue the phenotype, we inhibited cell proliferation by crossing *Wrn^-/-^*mice with *p53^S/S^* mice. The occurrence of NTDs in *p53^S/S^ Wrn^-/-^*mice was 35.2%, thus suggesting that the inhibition of cell proliferation through a Wrn defect partially rescued the exencephaly phenotype in p53^S/S^ mice. We also report that p53S decreased expression of UTX at mRNA and protein level via increasing Xist transcript, result in high female-specific H3K27me3 expression and repressed Mash1 transcription, which facilitating abnormal proliferation, differentiation, and apoptosis, result in the mis-regulation of neurodevelopment and neural tube defects (NTDs).

## Introduction

Neurulation is a defining feature of vertebrate development and is essential for proper nervous system development and embryonic viability 1-4. Failures in neural tube closure, known as neural tube defects (NTDs), are highly prevalent congenital malformations. NTDs are classified on the basis of the region of the neural tube affected and include exencephaly (cranial), craniorachischisis (trunk), and spina bifida (posterior)^5^. Despite the clinical importance of NTDs, their causes are poorly understood.

The neural tube is formed by thickening of the dorsal surface ectoderm, which folds and joins at the midline. Neural tube closure is complex and requires tightly regulated coordination of numerous processes, including polarized apical constriction, proliferation and apoptosis 6, 7.Proliferation of neuroepithelial cells occurs before closure, thus increasing the number of cells within the neural tube. Neuroepithelial cells then give rise to progenitors that can either continue through the cell cycle or generate neural cells, first through neurogenesis and later by gliogenesis^8^. Maintenance of adequate proliferation in the neuroepithelium appears to be crucial for closure, particularly in the cranial region. The molecular mechanisms regulating the temporal sequence of neuroepithelial cell proliferation are poorly understood.

There is growing evidence that the tumor suppressor p53 (Trp53), which is known to prevent tumor development by promoting growth arrest and apoptosis, also functions as a regulator of cell differentiation 9, 10. Several mouse studies have shown that some p53 null embryos display diverse craniofacial abnormalities such as exencephaly^11^, ^12^. Furthermore, in situ hybridization has shown that p53 is expressed in the neural tube and in neural crest cells 13, 14. In neural progenitor cells and gliomas, p53 directly represses Id2, thereby inhibiting cell proliferation and self-renewal. In addition, other studies have shown that abnormal activation of p53 signaling leads to embryonic developmental failure 15-17. These studies support the notion that p53 not only functions as a tumor suppressor but also plays an essential role in embryonic development. However, its precise roles during embryogenesis remain to be elucidated.

p53 is the most frequently mutated tumor suppressor gene involved in human tumorigenesis. Mutant p53 is thought to contribute to carcinogenesis through the acquisition of gain of function properties. It is highly likely that p53 mutations would result in impaired development. Indeed, more recently, p53 has been shown to have a gain of function mutation in CHARGE syndrome, a multiple anomaly disorder in humans that arises from mutations in the *CHD7* gene. In that study, p53^25, 26, 53, 54^ mutant protein has been shown to stabilize and hyperactivate wild-type p53, which then inappropriately induces its target genes and triggers cell cycle arrest or apoptosis during development^17^. However, very few studies have used mouse models to investigate p53 mutant function in embryonic development.

The p53^N236S^mutation (p53^N239S^ in humans, referred to here as p53S) has been reported as a somatic mutation in 35 tumor cases in IARC databases. Our previous study has described this p53 mutation in mouse MEFs. The mutation has been found in three independent tumorigenic mouse cell lines that use the alternative lengthening of telomeres (ALT) mechanism to maintain cell proliferation^18^. The p53S mutant has lost its DNA binding ability and consequently its ability to regulate cell cycle arrest and apoptosis in response to irradiation. In addition, p53S cross-talk with H-RasV12 reduces the cellular stress response to oncogenic signals, thus facilitating cell growth and tumorigenesis.

In the present study, we identified the role of p53S in mammalian development by producing a *p53^S/S^* knock-in mouse model. The p53S mutation led to decreased neuroepithelial differentiation and apoptosis, increased neuroepithelial proliferation during neurulation, and failure in neural tube closure. Strikingly, we found that knockout of the DNA helicase Wrn inhibiting cell proliferation could partially rescue the phenotypes in *p53^S/S^* embryos, thus demonstrating that cell proliferation contributes to phenotypes resulting from p53S mutation.

In this context, we propose that p53S is involved in neural tube closure through regulating H3K27me3 expression to tightly coordinate neuroepithelial cell proliferation, differentiation and apoptosis, during neurulation.

## Results

### p53S is a novel mutant with defects in neural tube

**Figure 1:**
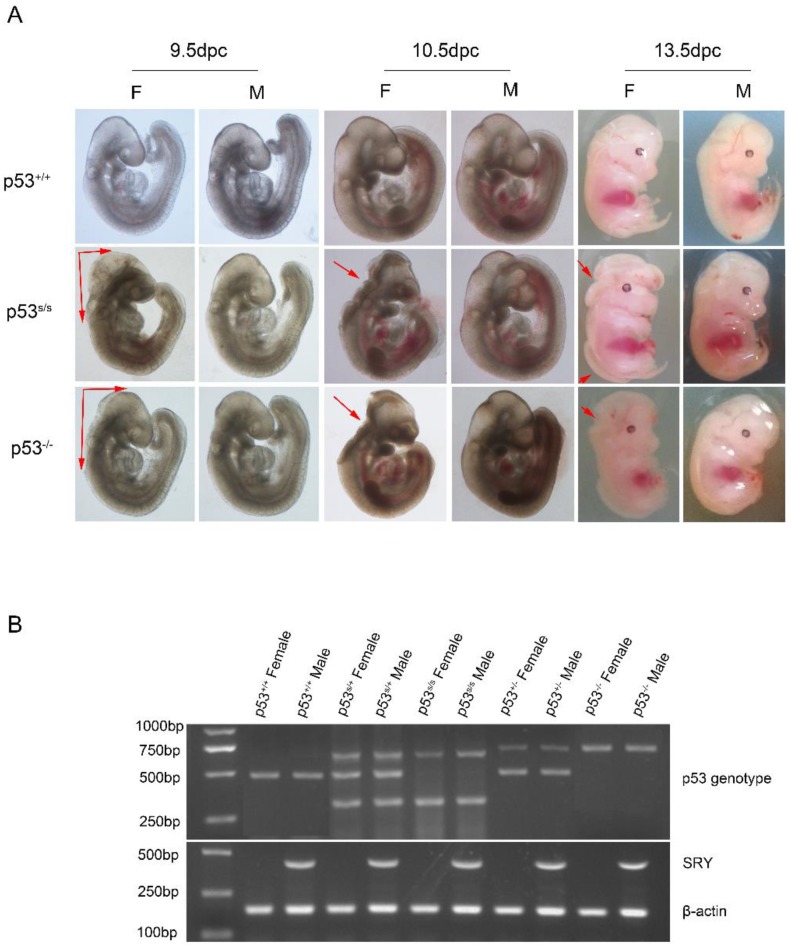
** p53S mutation leads to failure of neural tube closure in female embryos.** (A) Morphology of WT, *p53^-/-^*, *p53^S/S^* embryos at different time points (9.5dpc, 10.5dpc and 13.5dpc). Red arrowheads indicate exencephaly and spina bifida. (B) PCR genotyping and sex determination results.

The *p53^S/S^* knock-in mice were generated by a commercial service. The details are described elsewhere 19. We used a three-primer genotyping protocol to distinguish between WT and the *p53^S/S^*homozygote mutant in a single PCR reaction (Fig. 1B). PCR analysis revealed that among149 live progeny from *p53^S/+^*intercrosses, 42 were wild type, 84 were *p53^S/+^*, and 23(15.4%) were *p53^S/S^*mice (Table 1). This distribution of progeny is different from the expected Mendelian ratio of 1:2:1. To maximize the generation of *p53^S/S^*mice, we chose to use another mating strategy. We crossed *p53^S/S^* with *p53^S/+^*and obtained 41.0% (68/166) homozygous mutants, a value less than the anticipated value of 50% (Table 2). These surprising results suggested that a fraction of the *p53^S/S^*mice either do not survive during gestation or die in the perinatal period before weaning.

To address whether *p53^S/S^* embryos developed abnormally and died, timed embryos analysis were performed. We recovered homozygous mutant embryos from E9.5-E13.5 with expected Mendelian frequency. A total of 32 of 139 (23 %) *p53^S/S^* mutant embryos were observed from *p53^S/+^*intercrosses.

**Figure 2:**
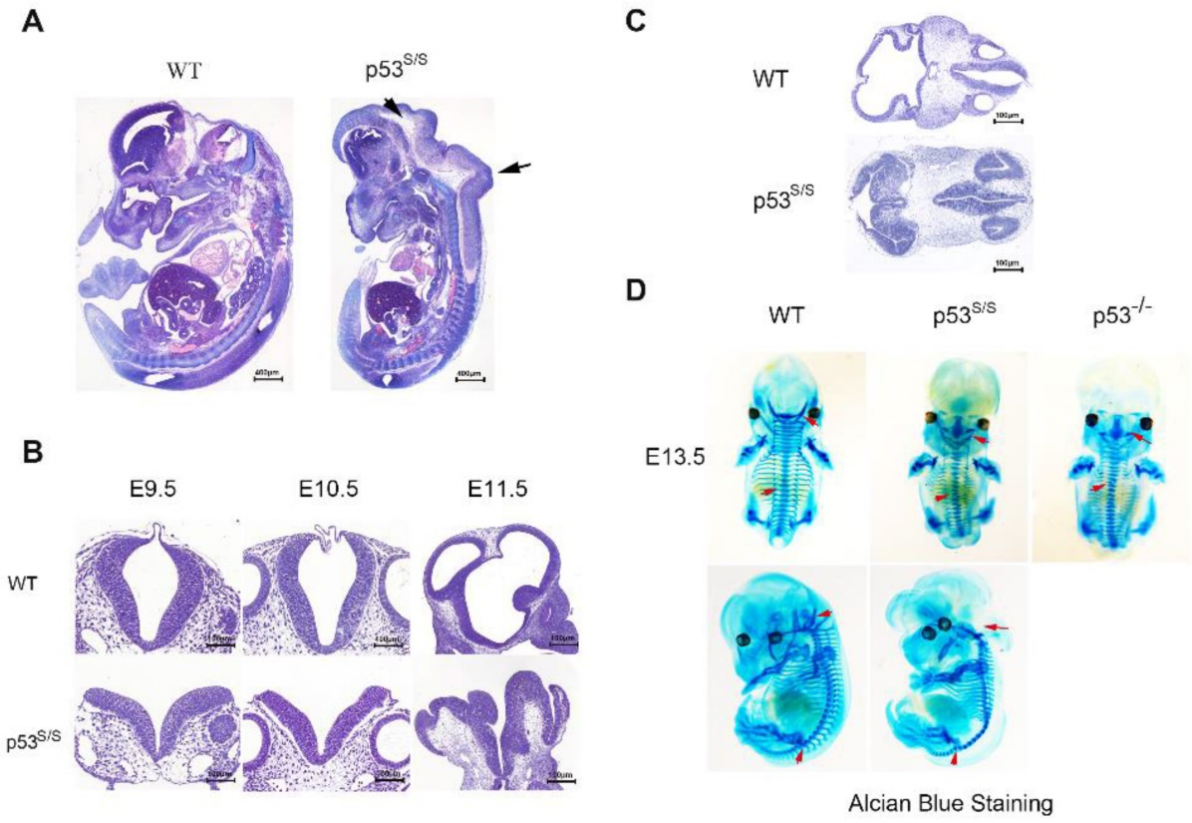
** Characterization of the neural tube opening phenotype and axial skeletal malformation. (A)** H&E staining of sagittal sections of E13.5 embryos showed neural tube opening from the midbrain to hindbrain of p53^S/S^ embryos (black arrowheads). **(B)** H&E stained transverse head sections of WT and p53^S/S^ embryos at different stages of development. Scale bar, 400µm **(C)** Collapse of telencephalic and fourth vesicles of p53^S/S^ versus WT embryos at E11.5. Lines on the left panel illustrate the plains of sectioning. **(D)** Staining with Alcian Blue to visualize abnormal fused vertebral bodies and absent foramen magnum region as well as curled tail in p53^S/S^ NTDs (red arrowheads).

Our analysis from E9.5 and E10.5 shows that the majority of *p53^S/S^* embryos exhibit open neural tube defects (NTDs) with exencephaly; at E13.5, some exencephalic *p53^S/S^*embryos also exhibited spina bifida; whereas some mutant embryos have closed neural tubes (Fig. 1A). The neural tube was open throughout the remainder of development, resulting in large open anterior neural tubes encompassing the mid and hindbrain, with variable extension into the forebrain region and the spinal cord at E11.5 (Fig 2C). Serial coronal and transverse sections revealed severe brain compression and the collapse of ventricles in *p53^S/S^* embryos (Fig 2A-2B).

### Gender Bias in NTD *p53^S/S^*Mice

We also noted a skewed distribution ratio of females to males among the p53 mutant mice at weaning. The ratio of live-born female to male offspring was 1:3.8. Therefore, we used PCR to ascertain the sexes of *p53^S/S^*embryos. Of 352 female *p53^S/S^* embryos examined, 241(68.5%) had exencephaly (Table 3). Exencephaly was primarily found in the female embryos, a result consistent with previous observations in numerous models 20, 21. Some laboratories have reported that a subset of female *p53^-/-^* mice to die in utero^12^, ^22^-^25^. Whereas other groups have found *p53^-/-^*mice to be born at close to the expected frequency^26^. The basis of the variable penetrance of the developmental phenotype in *p53^-/-^*mice may be due to strain-specific modifiers. In our crosses, we found the exencephaly frequency of 37.1% (48/132) in *p53^-/-^*female embryos under the same genetic background (C57BL/6 × 129SvEv) (Fig 1A). The 68.5% exencephaly frequency in female *p53^S/S^*embryo was much higher than that observed in *p53^-/-^* mice, suggesting that p53S is a new p53 gain of function mutation affecting regulation of NTDs 27.

The spinal column is often deformed in both human and animal models of NTDs^28^. Examination of the skeletons of late gestation *p53^S/S^*mice stained with alcian blue revealed several abnormalities. Alcian blue staining of the initial cartilage matrix showed abnormal spinal fusion in *p53^S/S^* and *p53^-/-^* NTDs (Fig 2D).

### p53S Promotes Neuronal Proliferation

**Figure 3:**
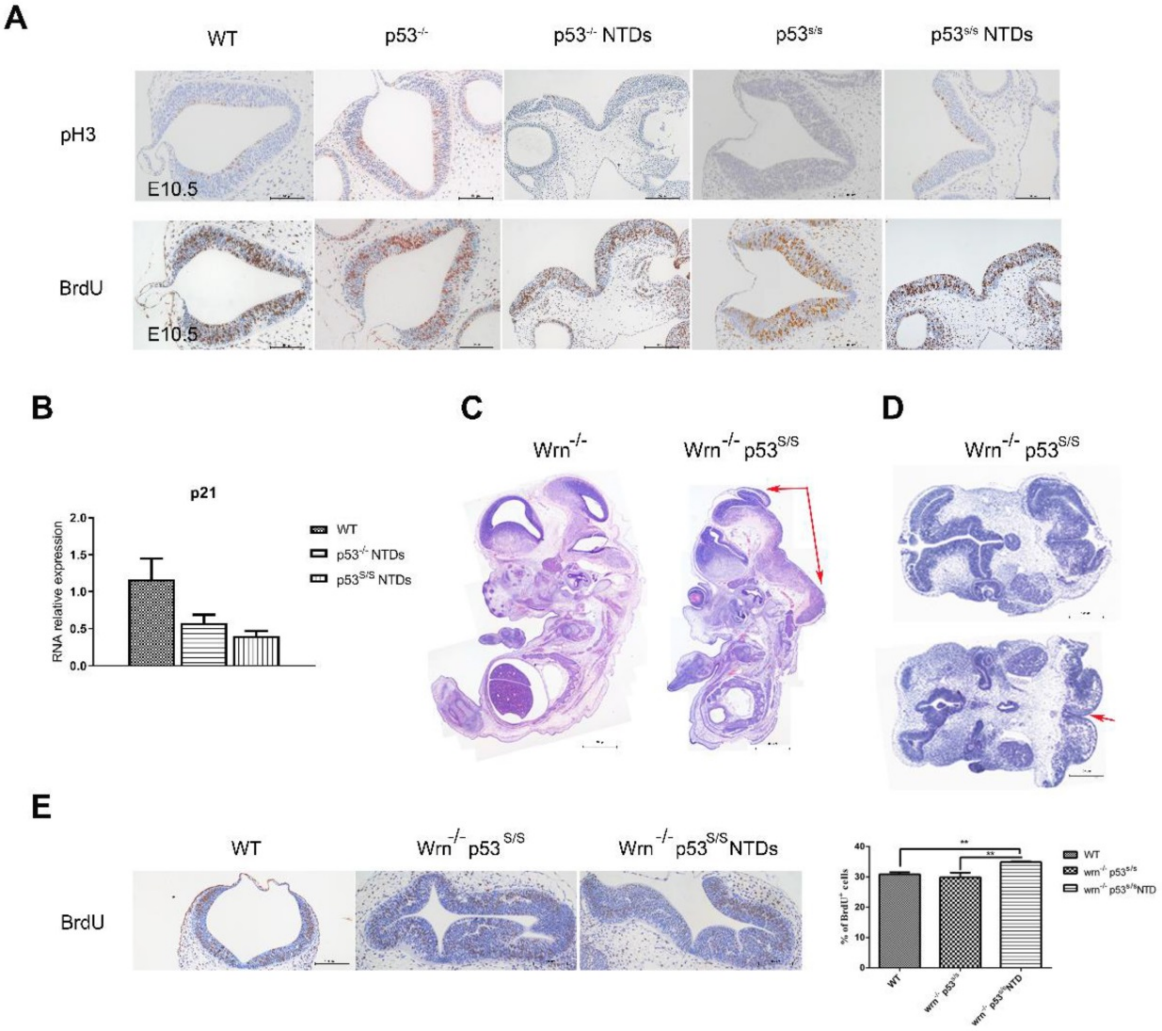
** Increased Proliferation of the Neuroepithelium in p53^S/S^ Embryos. (A)** Immunohistochemistry against PH3 and BrdU incorporation analysis, Scale bar, 100µm. (n = 3 embryos, three sections per embryo). *p < 0.05, **p < 0.005. Quantification of PH3-positive cells was calculated as the percentage of PH3-positive cells relative to the total number of neuroepithelial cells at E10.5. Quantification of BrdU-positive cells was calculated as the percentage of BrdU-positive cells relative tothe total number of neuroepithelial cells at E10.5. Error bars represent SD. (B) Real-time PCR detected p21 expression in WT, *p53^-/-^*NTDs and *p53^S/S^* NTDs**. (C)** H&E staining of sagittal sections of E13.5 embryos showed exencephaly (red arrowheads) in *Wrn^-/-^p53^S/S^* versus *Wrn^-/-^* embryos; Scale bar, 400µm. **(D)** Opening neural tube (red arrowheads) of *Wrn^-/-^p53^S/S^* at E10.5; Scale bar, 100µm.** (E)** BrdU incorporation analysis after a 2 hr pulse. Controls include WT, *Wrn^-/-^p53^S/S^* normal embryos and *Wrn^-/-^p53^S/S^* NTDs. Error bars represent SD (n = 3 embryos, three sections per embryo). *p < 0.05, **p < 0.005.

Neural tube closure is influenced by multiple cellular processes, including proliferation, differentiation, apoptosis, apical constriction, and patterning. We investigated whether there was any association between p53S and cell proliferation. Staining for phospho-histone H3 (p-H3) in hindbrain revealed a significant increase in the number of cells in M phase in p53 mutant opening neural tube than that in *p53^-/-^* and WT normal neural tube at E10.5(Fig 3A). To analyze the S phase, we performed bromodeoxyuridine (BrdU)-labeling experiments and found a significant increase in BrdU-positive cells at E10.5 in the exencephaly in *p53^S/S^*(Fig 3B). Our previous study found that p53S lost transcriptional regulatory function in to cell cycle regulator p21 regulated by wild type p53, thus promoted cell growth greatly 18. We performed quantitative PCR analysis on RNA harvested from the heads of female wild-type, *p53^-/-^*and *p53^S/S^* NTDs embryos and observed p21 mRNA level was down-regulated in *p53^-/-^*and *p53^S/S^* NTDs. These data revealed p53S enhanced abnormal cell proliferation via down-regulating p21 expression, result in neural tube defects.

To further investigate the effect of abnormal cell proliferation on neural tube defects, we inhibit cell proliferation in *p53^S/S^* mice. We cannot properly overexpress p21 in the *p53^S/S^* mice, thus chose to use another strategy. We inhibited cell proliferation through knockout *Wrn* gene. *Wrn* encodes Werner syndrome protein (WRN) with both DNA helicase and exonuclease activity. Mutations in the *Wrn* gene are associated with a progeroid syndrome in humans (Werner Syndrome, WS), which is characterized by accelerated aging, cellular senescence, genomic instability and so on. *Wrn^-/-^*mice exhibit many of the WS phenotypes including a decreased life expectancy, but do not show any signs of NTDs (Fig 3C). We crossed *Wrn^-/-^* and *p53^S/S^* mice to assess. We obtained 58/ 191(30.4%) homozygous mutant (*Wrn^-/-^p53^S/S^*) offspring from breeding *Wrn^-/-^p53^S/S^*males to *Wrn^-/-^p53^S/+^*female mice, 21/165(12.7%) *Wrn^-/-^p53^S/S^* offspring from *Wrn^-/-^p53^S/+^*males to *Wrn^-/-^p53^S/+^*female mice at the time of genotyping (Table 4, Table 5). The distribution of *Wrn^-/-^p53^S/S^*mice is still less than expected Mendelian frequency. Next, we investigated offspring at E9.5-E13.5. Of 68 female *Wrn^-/-^p53^S/S^* embryos examined, 24(35.3%) had exencephaly, a value less than the number of exencephalic *p53^S/S^* mice (68.5%)(Fig 3D, Table 3).

In addition, some *Wrn^-/-^p53^S/S^* embryos were dead before E9.5. The *Wrn^-/-^p53^S/S^* embryos are embryonic lethal might due to genomic instability. Transverse sections also showed severe brain compression and the collapse of ventricles in *Wrn^-/-^p53^S/S^* embryos (Fig 3D).

We also examined the state of cell proliferation in* Wrn^-/-^p53^S/S^* embryos with spina bifida (Fig 3E). There were also increases to 35% of BrdU^+^ cells at E10.5 in *Wrn^-/-^p53^S/S^* embryos with spina bifida than in the controls, but less than *p53^S/S^* embryos with exencephaly. NTDs phenotypes were partially rescued by inhibition of cell proliferation in *p53^S/S^* embryos, suggesting that p53S still gain new function of regulation of neural tube closure.

### p53S Reduces Neuronal Differentiation and Apoptosis

**Figure 4:**
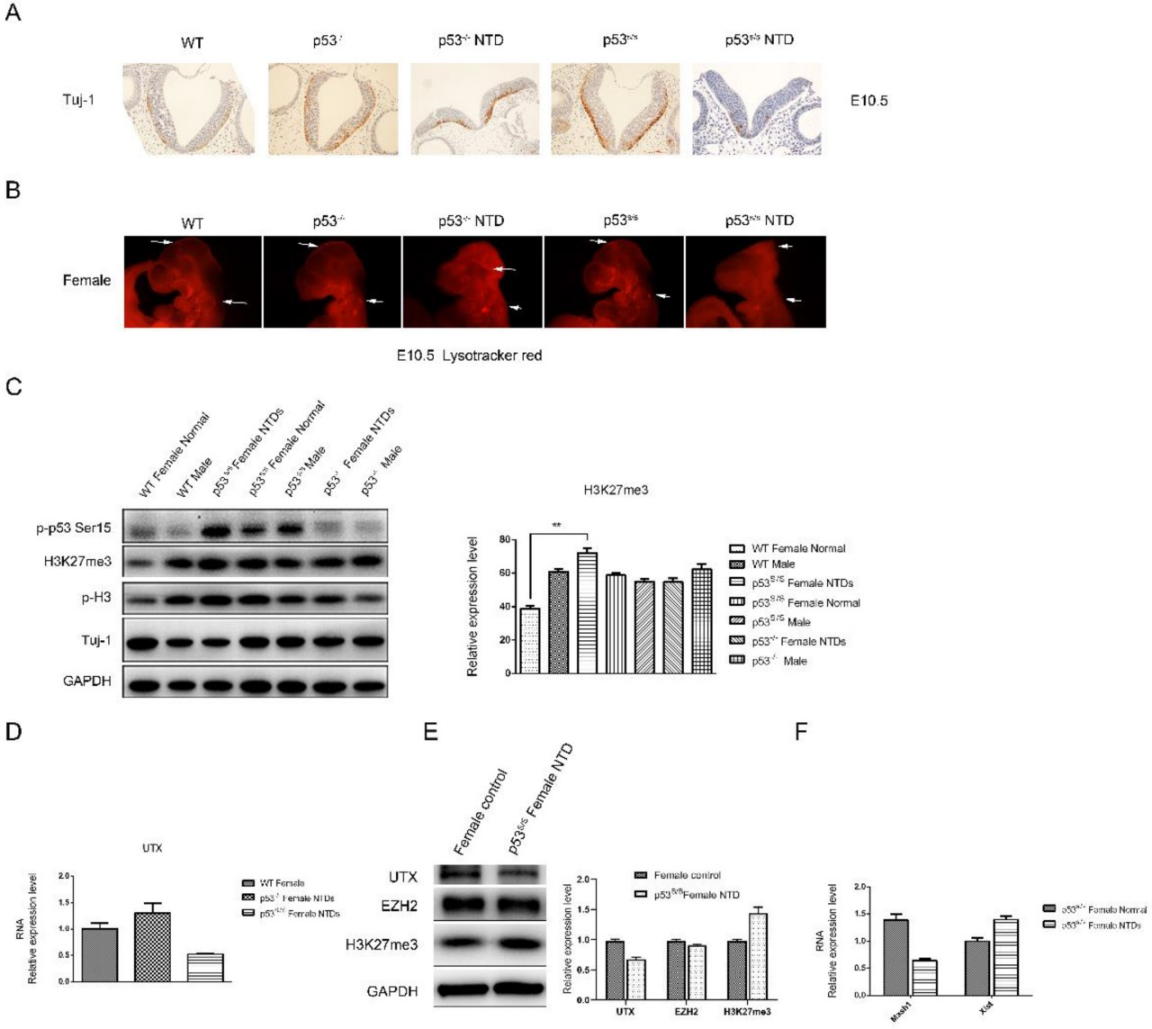
** Decreased Neural Differentiation and apoptosis of p53^S/S^ Embryos and increased expression of H3K27me3 in female p53^S/S^ NTDs embryos. (A)** Immunohistochemistry against Tuj-1 was used to identify neurons at E10.5. Neurons were stained with Tuj-1 (brown), a early post-mitotic neurons marker, Scale bar, 100µm. **(B)** LysoTracker staining was used to identify cell apoptosis at E10.5. The apoptosis cell were stained with LysoTracker red ( red ). Scale bar, 100µm. **(C)** The p-p53, H3K27me3, p-H3, Tuj-1analysis by Western blotting. Quantification of the Western blotting and statistical analysis. **(D)** The mRNA expression levels of UTX in WT, *p53^-/-^*NTDs and *p53^S/S^* NTDs measured by qRT-PCR. **(E)** The expression of UTX, EZH2, H3K27me3 analysis by Western blotting in Normal female embryo and *p53^S/S^* NTDs. **(F)** qRT-PCR detection of Mash1 and Xist in Normal female embryo and *p53^S/S^* NTDs.

We asked whether there were defects in neuronal differentiation in p53^S/S^ embryos. Tuj1 (acetylated β-III tubulin) marks early post-mitotic neurons, which are first seen at about E9.5. *p53^S/S^* embryos showed significantly decrease Tuj1^+^ cells compared with *p53^-/-^* and wild-type at E10.5 (Fig 4A). LysoTracker staining for dead cells (apoptosis cells) in whole-mount embryos showed a striking reduction in cell death in the hind and midbrain region of E10.5 embryos^8^ (Figure 4B). Together, these findings show that the p53S gain new function of leading to decreased neurogenesis and apoptosis during neurulation of the neural tube.

### p53S Increases Female-specific H3K27me3 Expression in NTDs

We checked p53S expression in *p53^S/S^* NTDs embryos and observed increased p-p53S protein expression in *p53^S/S^* embryos relative to WT or *p53^-/-^* embryos (Fig 4C). The cellular functions of p53 are largely controlled by phosphorylation event.

Histone modification has been known as an important component of embryo epigenetics. In embryonic stem cells (ESCs), many developmental genes exhibit 'bivalent state' marked by bivalent trimethylation of both histone H3-lysine 27 (H3K27me3) and H3-lysine 4 (H3K4me3) 29. Given that the H3K27me3 mark is important to repress many developmental genes in self-renewing ESCs, the removal of H3K27me3 in a cohort of tissue-specific genes is likely an important step in organogenesis^30^, ^31^. During the closure of neural tube, the demethylation of H3K27me3 was increased, which promotes the differentiation of neural stem cells. The total H3K27me3 level was detected by Western blot analysis (Fig 4C). High H3K27me3 level was detected in *p53^S/S^* NTDs embryos compared among female WT, *p53^-/-^* and* p53^S/S^* embryos at E10.5. While no change of H3K27me3 expression was in male WT, *p53^S/S^* and *p53^-/-^* embryos. Histone H3K27me3 mark is catalyzed by the histone methyltransferase enzyme, enhancer of zeste homolog 2 (EZH2); can be erased by H3K27me3-specific demethylases, UTX (also called lysine-specific demethylase 6A [KDM6A]). We examined UTX and EZH2 expression in *p53^S/S^* NTDs embryos and observed decreased UTX expression in *p53^S/S^* NTDs embryos relative to *p53^S/S^* normal embryos, while EZH2 expression no change (Fig 4E). UTX, X-linked H3K27me3 demethylase, is encoded on the X chromosome but escapes X inactivation in females and is ubiquitously expressed. In males, its homolog on the Y chromosome, UTY, could compensate for UTX loss during development. UTX null embryos had female-specific neural tube closure defects^32^. We performed quantitative PCR analysis and found down-regulated UTX and up-regulated Xist mRNA expression in *p53^S/S^* NTDs (Fig 4D, 4F). These data suggested p53S decreased expression of UTX at mRNA and protein level via increasing Xist transcript, resulting in high female-specific H3K27me3 expression and neural tube defects.

Next, we observed a significantly decrease in Mash1 transcripts (Fig 4F). Mash1, a well-known activator-type bHLH gene, directly promoting the differentiation in neural progenitors. These results suggested that high female-specific H3K27me3 expression repressed Mash1 transcription and neural differentiation.

Also, we observed reduced Tuj-1 and increased p-H3 expression in the *p53^S/S^* NTDs embryos relative to WT or *p53^-/-^* female embryos, which is consistent with the observation of histology (Fig 4C).

## Discussion

Although significant progress has been made in understanding the function of mutant p53 in cancer, its role during embryogenesis is far less clear. In this study, we used *p53^S/S^*knock-in mouse embryos to study the involvement of the p53 N236S mutation in neural tube closure, a key process during embryogenesis.

Surprisingly, we found that the p53S lead to embryonic and peri-natal lethality. The phenotype of homozygous *p53^S/S^* embryos is neural tube opening, such as exencephaly and spina bifida, which is observed beginning at E9.5. 68.5% p53 homozygous mutant female mice had exencephaly in the present study. Previously, exencephaly in 23% of females has been observed in p53 homozygous KO mice^11^. In our study, we found that 37.1% *p53^-/-^*female mice exhibited exencephaly under the same genetic background as that in* p53^S/S^*. Together, these data identify a novel function of p53S in neural tube closure.

NTDs in *p53^S/S^* mutant mice occurred predominantly in females, a result similar to the phenotypes observed in *p53^-/-^* mice. A female sex bias for NTDs has also been reported in humans^33^. The female bias in NTDs in *p53^-/-^* mice has been attributed to the presence of an extra X chromosome 21. The molecular mechanism by which the number of X chromosomes influences NTDs is not well understood. In humans, the p53 mutant has also been reported to have sex-specific effects on longevity and cancer rates 34, 35.

It is now generally accepted that cell proliferation, differentiation and apoptosis cannot occur simultaneously; these events are mutually antagonistic. Near the time of neural tube closure, some of the cells in the neural tube escape from the cell cycle and enter neuronal differentiation^36^, thus suggesting that a balance between continual neuronal cell differentiation and proliferation is crucial for the proper closure of the neural tube^6^. There is increasing evidence that p53 regulates neural differentiation, although conflicting results have been reported 37-40. Whereas several studies have shown that p53 inhibits neuronal lineage, other studies have reported that p53 plays no role in neuronal differentiation 41-43.

Our in vivo detection of early neuron marker Tuj1 with immunochemistry showed that the p53S led to decreased neuronal differentiation (Fig 4A). LysoTracker staining in whole-mount embryos showed that p53S reduced neuronal apoptosis (Fig 4B). Let-7, one of the earliest miRNAs discovered, has also been proposed to regulate the timing of differentiation of several lineages in C. elegans^44^. Moreover, let-7 has recently been shown to regulate the timing of the neurogenic to gliogenic transition during mouse neural development 45, 46. MiR-302 and let-7 have opposite functions, and miR-302 loss leads to accelerated differentiation 8. p53S might act on these pathways, thus regulating the timing of cell fate decisions. The coordinated action of these pathways may help to fine tune p53S in cell differentiation.

Rapid cell proliferation is necessary to generate the large number of neural cells required for the development of the neural tube. This rapid cell cycling is tightly regulated by the signaling molecules FGF, Wnt and TGF-β, which are secreted by the neuroepithelium^47^. Interestingly, high concentrations of Shh have been reported to inhibit local cell proliferation 48. Slit/Robo1 signaling up-regulates ventral neural tube marker Shh, thus causing abnormal cell proliferation phenotypes 49. Abnormal cell proliferation has been observed in embryonic neural cells in which p53 function is inhibited by the expression of oncogenic viruses 50. By detecting proliferation markers BrdU, we showed that the p53S mutant led to increased cell proliferation at E9.5 and E10.9.We thus speculate that new target genes of p53S may play a central role in neural development.

Future studies of the function of the p53S mutation are predicted to provide insight into whether p53S modifies the expression of any genes on the X chromosome. H3K27me3, a signal for gene silencing, plays an important role during the development of embryonic neural tube^51^. Western blotting showed that high H3K27me3 expression and p-p53S in the *p53^S/S^* NTDs embryos, suggesting p53S induced female-specific H3K27me3 overexpression, lead to NTDs. Furthermore, we demonstrate that UTX is required for H3K27me3 demethylation during neural tube closure. Lee et al reported a requirement for UTX in cardiac development due to its ability to activate cardiac-specific genes in both a demethylase-dependent and -independent manner 52. Our analysis extends this conclusion and suggests that UTX can be regulated by p53S during development evidenced by observed *p53^S/S^* NTDs.

From our results, we propose that the *p53^S/S^* mouse model explains the gain of function of the p53S in neural tube development. p53S induces female-specific H3K27me3 expression via decreasing UTX, which inhibited neural differentiation and apoptosis and activated neural proliferation during the onset of neural tube development, and disturbance at this developmental stage creates neural tube defects.

## Materials and Methods

### Generation of p53^S/S^ knock-in mice

The *p53^S/S^* knock-in mice were established by a commercial service (inGenious Targeting Laboratory, Inc., NY). The details are described in another paper 19. All the mice involved in procedures were in line with the Guide for the Care and Use of Laboratory Animals. Animal experiments and protocols were approved by the Animal Care and Use Committee of Kunming University of Science & Technology.

### Genotyping and sex determination by PCR

Genotyping was performed by using a three-primer PCR assay of genomic DNA prepared from either tail clippings of pups or embryonic tissues. A 3′primer (Neo11-R primer) downstream of the neo cassette was combined with two 5′primers located downstream of the neo cassette(Neo10-F) and in the loxp site (F3) to generate different-sized PCR products from the wild type allele (458 bp) and N236S mutant (294 bp, 634 bp). Primer sequences are as follows: Cre primers: 5'-TCCAATTTACTGACCGTACACCAA-3'; 5'-CCTGATCCTGGCAATTTCGGCTA-3'and the p53S primers: NeoF: 5'-CTGCACCCTACGAGAACTGACTT-3'; NeoR: 5'-GGGATGAAGTGATGGGAGCTAG-3'; and F3: 5'-GCATAAGCTTGGATCCGTTCTTCGGAC-3').

The PCR to determine embryo sex was done with male-specific SRY primer. PCR of the male will generate a 420 bp band and female no band. β-actin provides a PCR control. Primer sequences are as follows:

SRY primers: 5'-ATTTTTAGTGTTCAGCCCTAC-3'; 5'-CTACTCCAGTCTTGCCTGTAT-3'; β-actin primers: 5'-TTCTTCTGCCGTTCTCCCAT-3'; 5'-GCTTTGTCACACGAGCCATTG-3'.

PCR was run for 35 cycles (15 seconds at 95, 30 seconds at 60°C, 60 seconds at 72°C). PCR products were visualized on a 2% agarose gel stained with ethidium bromide.

### Analysis of embryos

Crosses of heterozygous mice or heterozygous with homozygous mice were performed using mice that were backcrossed with C57BL/6 mice. The day on which the vaginal plug was detected was considered to be day 0.5 post-coitum (designated E0.5). Pregnant females were sacrificed at different day's post-coitum. Embryos were dissected from the uterus in PBS and examined for external abnormalities, including exencephaly, spina bifida.

### Histology

Embryonic samples from timed matings were fixed in 4% paraformaldehyde (PFA) at 4°C overnight, dehydrated in graded solutions of alcohol, cleared in xylene, embedded in paraffin, and cut into 3μm thick sections before hematoxylin and eosin (H&E) staining.

Sections were blocked with 5% goat serum, incubated in primary antibody in blocking solution at 4ºC overnight. pH3 (1:500, Cell Signaling, Tuj1 (1:1, 000, Covance MMS-435P-250).

For whole-mount LysoTracker evaluation of cell death, embryos were dissected in PBS, incubated in LysoTracker Red (1:5000) (Life Technology L7528) for 45 min at 37ºC. Samples were then washed three times in PBS and imaged.

### Western blotting

Brain tissue lysates were prepared at E10.5. Samples with equal concentrations of protein (25 ug/lane) were electrophoresed. The samples were then transferred to a methanol-preactivated PVDF membrane (Roche, Germany) and blocking was done for 60 minutes at room temperature using a solution of 5% w/v dried non-fat milk in TBS plus 0.1% Tween-20. Antibodies used for western blot were anti-phospho-p53 (Ser15) (1:500, Cell Signaling), anti-H3K27me3 (1:1000, Cell Signaling), anti-pH3 (1:500 Cell Signaling), anti-Tuj1 (1:1, 000 Covance MMS-435P-250), anti-EZH2 (1:1, 000 Millipore), anti-UTX (1:500 Abcam).

### Alcian blue staining

For cartilage staining, E13.5 embryos were fixed in Bouin's solution. After washing in 70% ethanol, the embryos were equilibrated in 5% acetic acid. The embryos were stained in 0.05% Alcian blue in 5% acetic acid for 2 h, followed by two washes in 5% acetic acid and then cleared in methanol for 2 h before stored in 1:2 mixture of benzyl alcohol and benzyl benzoate.

### RNA Isolation and qRT-PCR

Total RNA was isolated from the heads of WT and p53S/S embryos using Trizol (Invitrogen) and reverse-transcribed using the SuperScript III First-Strand Synthesis System (Invitrogen). RNA quantity was determined by agarose gel electrophoresis and by spectrophotometry. Quantitative real-time PCR was performed using SYBR (Takara Bio, Japan) in a 10uL system on an ABI PRISM 7300 sequence detector system (Applied Biosystems). The levels of mRNA were normalized against the levels of β-actin. The Primer sequences are as follows: p21 primers: 5'-CCAGGCCAAGATGGTGTCTT-3'; 5'-TGAGAAAGGATCAGCCATTGC-3'; Mash1 primers: 5'-AAGAGCTGCTGGACTTTACCAACTG -3'; 5'-ATTTGACGTTGGCGAGA-3'; β-actin primers: 5'-AGAGGGAAATCGTGCGTGAC-3'; 5'-CAATAGTGATGACCTGGCCGT-3'. The relative expression level of each mRNA was analyzed by the 2^-△△Ct^ method. All experiments were repeated three times.

### Statistical Analysis

Student′s t-tests were used to assess the significance of the differences between two groups of data (p < 0.05 is deemed significant). The Chi squared tests were used to evaluate the significance of differences between the expected and observed genotype distributions.

## Figures and Tables

**Table 1:** Genotype distribution of embryos from intercrosses of p53^S/+^ mice

Expected	*p53^S/+^♂ x p53^S/+^♀*			p Value
	*p53^S/S^*	*p53^S/+^*	*WT*	
	25%	50%	25%	
E8.5-E13.5	32	72	35	0.5745
P20	23	84	42	<0.01

**Table 2:** Genotype distribution of embryos from intercrosses of p53^S/S^ and p53^S/+^ mice

Expected	*p53^S/S^♂ x p53^S/+^♀*		p Value
	*p53^S/S^*	*p53^S/+^*	
	50%	50%	
E8.5-E13.5	135	142	0.6741
P20	68	98	0.0199

**Table 3:** The incidence of Exencephaly in *p53^-/-^*, *p53^S/S^* and *Wrn^-/-^p53^S/S^* female embryos.

Genotype	Exencephaly Female	Total Female	%
*p53^-/-^*			23%^11^
*p53^S/S^*	241	352	68.5%
*Wrn^-/-^p53^S/S^*	24	68	35.2%

**Table 4:** Genotype distribution of embryos from intercrosses of *Wrn^-/-^*p53^S/+^ mice

Expected	*Wrn^-/-^p53^S/+^♂ x Wrn^-/-^p53^S/+^♀*			p Value
	*Wrn^-/-^p53^S/S^*	*Wrn^-/-^p53^S/+^*	*Wrn^-/-^*	
	25%	50%	25%	
E8.5-E13.5	43	94	35	0.627152
P20	21	108	36	<0.01

**Table 5:** Genotype distribution of embryos from intercrosses of *Wrn^-/-^*p53^S/S^ and *Wrn^-/-^*p53^S/+^ mice

Expected	*Wrn^-/-^*p53^S/S^♂ x *Wrn^-/-^*p53^S/+^♀		p Value
	*Wrn^-/-^*p53^S/S^	*Wrn^-/-^*p53^S/+^	
	50%	50%	
E8.5-E13.5	89	99	0.465803
P20	58	133	<0.01
